# Biocompatibility and Toxicity of Poly(vinyl alcohol)/N,O-Carboxymethyl Chitosan Scaffold

**DOI:** 10.1155/2014/905103

**Published:** 2014-09-15

**Authors:** Tunku Kamarul, G. Krishnamurithy, Noman D. Salih, Nurul Syuhada Ibrahim, Hanumantha Rao Balaji Raghavendran, Abdul Razzaq Suhaeb, D. S. K. Choon

**Affiliations:** Tissue Engineering Group (TEG), National Orthopaedic Centre of Excellence in Research and Learning (NOCERAL), Department of Orthopaedic Surgery, Faculty of Medicine, University of Malaya, 50603 Lembah Pantai, Kuala Lumpur, Malaysia

## Abstract

The in vivo biocompatibility and toxicity of PVA/NOCC scaffold were tested by comparing them with those of a biocompatible inert material HAM in a rat model. On Day 5, changes in the blood parameters of the PVA/NOCC-implanted rats were significantly higher than those of the control. The levels of potassium, creatinine, total protein, A/G, hemoglobulin, erythrocytes, WBC, and platelets were not significantly altered in the HAM-implanted rats, when compared with those in the control. On Day 10, an increase in potassium, urea, and GGT levels and a decrease in ALP, platelet, and eosinophil levels were noted in the PVA/NOCC-implanted rats, when compared with control. These changes were almost similar to those noted in the HAM-implanted rats, except for the unaltered potassium and increased neutrophil levels. On Day 15, the total protein, A/G, lymphocyte, monocyte, and eosinophil levels remained unaltered in the PVA/NOCC-implanted rats, whereas urea, A/G, WBC, lymphocyte, and monocyte levels remained unchanged in the HAM-implanted rats. Histology and immunohistochemistry analyses revealed inflammatory infiltration in the PVA/NOCC-implanted rats, but not in the HAM-implanted rats. Although a low toxic tissue response was observed in the PVA/NOCC-implanted rats, further studies are necessary to justify the use of this material in tissue engineering applications.

## 1. Introduction

Scaffolds play a unique role in tissue regeneration and repair. In tissue engineering applications, an ideal scaffold may elicit a minimal degree of sublethal toxicity [1]. Poly(vinyl alcohol)/NOCC-based hydrogel is an organic material, with NOCC being a derivative of chitosan, a natural polymer from renewable resources such as shell of shellfish, and PVA being a water-soluble, biocompatible [2], and biodegradable polymer [3]. Due to its desirable characteristics such as nontoxicity, anticarcinogenicity, and appropriate mechanical properties [4, 5], poly(vinyl alcohol) (PVA) is a widely used polymer and is well-known for its excellent weight-bearing properties and compatibility. Few studies have shown that hydrogels prepared using PVA showed good biomechanical properties and in addition they are considered as a suitable candidate to prepare highly porous scaffolds when combined with agents like sucrose, polyethylene glycolic acid. Some of the biomedical applications of PVA include drug delivery, wound dressings, dialysis membranes, and cardiovascular devices [6–8]. In our previous studies, the viscoelastic potential of PVA/NOCC was demonstrated. PVA/NOCC not only exhibited good biocompatibility in vitro, but was also found to possess many preferable scaffold characteristics for tissue engineering applications [8, 9]. However, the in vivo biocompatibility of this material has not yet been demonstrated, which is particularly important in elucidating the inflammatory responses when implanted in preclinical animal models.

In the present study, we examined and compared the in vivo biocompatibility of a synthetic scaffold and a well-known biological scaffold of human amniotic membrane (HAM) during the early phase of implantation in rats. HAM is found in the lining of the placenta and was used in this study for comparison [10]. A toxicological evaluation was performed to demonstrate the systemic safety of both of the biomaterials.

## 2. Experimental Procedure

### 2.1. Preparation of PVA Hydrogels

**Figure 1:**
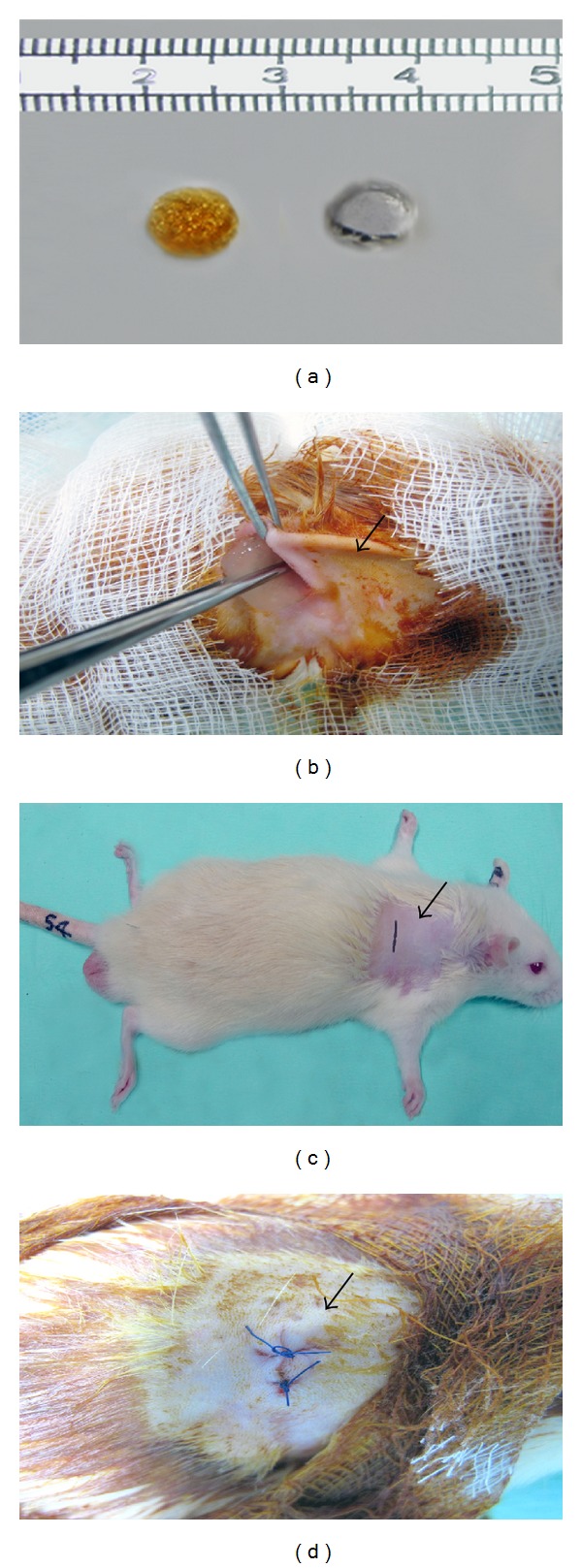
(a) Hydrogel and silicone discs. (b) The fur is cleared and marked incision area indicated by arrow. (c) Arrow indicates the representative animal and site of material implanted. (d) Representative photograph for site of implantation after suturing (arrow).

PVA-117 (Mw = 74,000 g/mol) was obtained from Kuraray Co. Ltd, Japan, and NOCC was obtained from the Standards and Industrial Research Institute (SIRIM), Malaysia. The porous hydrogel was prepared by blending PVA with NOCC at a PVA : NOCC ratio (w/v) of 20 : 5. PVA/NOCC was prepared using 20% PVA in distilled water. The polymer solutions were then cast into cylindrical molds and physically cross-linked by irradiation at 50 kGy. The hydrogels were frozen at −80°C for 24 h prior to lyophilization and subsequently cut into discs (Figure 1(a)).

### 2.2. Preparation of HAM Scaffold

The procedure for HAM scaffold preparation was approved by the Medical Ethics Committee of University of Malaya Medical Centre, reference number 612.56. Briefly, a total of six HAMs were obtained after informed consent from individuals who underwent elective cesarean sections. The selection criteria ensured that only donors who were seronegative for human immunodeficiency virus, human hepatitis B and C viruses, and syphilis were allowed to donate the tissues. The placenta tissues were placed in a sterile dish and washed under running water. The HAMs were peeled off carefully from the rest of the placental mass. Blood clots on the surface were washed with running water and subsequently with copious amounts of sterile saline. The HAMs were then immersed in saline and stored at 4°C overnight. After that, the HAMs were processed using sterile distilled water and rigorously shaken (100 rpm) for 10 min, followed by further washing in 0.05% sodium hypochlorite bath and gentle shaking (60 rpm) for another 10 min. Subsequently, the HAMs were washed thrice in sterile saline solutions for a period of 20 min each before being transected and subjected to air drying (AD) [10, 11].

### 2.3. Subcutaneous Implantation of the Biomaterials

The rats used in this study were adult male rats (Sprague-Dawley) weighing 250–300 g, which were maintained under light-dark cycle (12/12 h) and provided with food and water* ad libitum*. All surgical procedures involving animals were approved by the Animal Care and Use Committee (ACUC) of Faculty of Medicine, University of Malaya. The test materials PVA/NOCC were prepared as sterilized cylindrical discs of 8 mm diameter and 2 mm thickness, whereas the HAM was prepared as 30 mm square piece.

### 2.4. Description of the Implantation Procedure

The implantation procedure was performed under general anesthesia (80 mg/kg of ketamine and 5 mg/kg of xylazine administered through intramuscular (IM) injection) by making a transversal incision in the lumbar-sacral region (Figures 1(b)–1(d)).

### 2.5. Tissue Response Evaluation (Histology)

All animals were euthanized with intramuscular injections of pentobarbital. The subcutaneous tissues surrounding the implanted discs were carefully removed and fixed in 10% buffered formalin for 72 h. The obtained samples were dehydrated in sequential ethanol and Hemo-De solutions, embedded in paraffin, and cut into 5 *μ*m thick sections using a tabletop microtome (Thermo Scientific, USA). The sections were subjected to standard hematoxylin and eosin (H&E) staining procedure for histological examination. Briefly, the sections were washed and stained with hematoxylin for 5 min, transferred to cleadite (30 s) and then to bluing agent (30 s), rinsed, and stained with eosin for 1 min. Following dehydration, the sections were cleared and cover-slipped and viewed under microscope (Nikon, USA).

### 2.6. Complete Blood Count and Clinical Chemistry

In general, the animals were euthanized by carbon dioxide inhalation. Blood samples (approximately 3 mL of blood obtained by employing the cardiac puncture method) were obtained from all the animals in each group for each time point to have adequate volumes for all the analyses. A small portion of the whole blood (10–30 *μ*L) was used for performing the complete blood count and the remaining sample was used for serum separation. Thus, all the clinical chemistry and hematology data reported are the average values for the indicated time point within a group. The analyses carried out included the renal function test (potassium, urea, creatinine, and total protein), liver function test albumin : globulin (A/G), alkaline phosphatase (ALP), alanine and aspartate transaminases (AST, ALT), and gamma-glutamyl transferase (GGT), and complete blood count (hemoglobin, erythrocytes, white blood cells (WBC), platelets, neutrophils, lymphocytes, monocytes, and eosinophils). All the blood samples were subjected to complete routine chemistry and hematology tests using an autoanalyzer (Dimension Vista 1500, USA).

### 2.7. Immunohistochemistry

The skin samples were examined for anti-CD 68 antibody by employing immunocytochemistry staining using mouse monoclonal antibody against CD 68 (Abcam Plc., Cambridge, UK), according to the protocol provided by Dako Cytomation (Glostrup, Denmark). Briefly, the samples were rinsed with phosphate buffered saline (PBS) and fixed with methanol for 15 min. Then, the samples were treated with 0.03% hydrogen peroxide for 5 min and incubated with mouse anti-rabbit anti-CD 68 antibody for 30 min at 1 : 100 dilutions and then with peroxidase-labeled polymer conjugated to goat anti-mouse immunoglobulin for another 30 min. After washing with Tris buffered saline, the samples were incubated with substrate buffer containing 3,3-diaminobenzidine (DAB) chromogen, counterstained with hematoxylin, and mounted with mount solution. The specificity for CD 68 was confirmed, which exhibited reactivity for rat tissue in both frozen and paraffin-embedded samples. The stained sections were photographed using Nikon E200 (Tokyo, Japan).

### 2.8. Statistical Analysis

All data are presented as means ± standard deviations. Statistical analysis was performed using SPSS (version 17). Post hoc analyses were carried out using least significant difference (LSD) corrective method, and one-way ANOVA was deemed significant if *P* < 0.05.

## 3. Results

On day 5 (Table 1), when compared with the control group, the PVA/NOCC-implanted rats showed significant changes (*P* < 0.05) in all parameters, except for albumin, globulin, erythrocyte, platelet, and lymphocyte levels. On the other hand, the HAM-implanted rats showed significantly altered levels of urea, hepatic markers, neutrophils, eosinophils, and lymphocytes, when compared with the control (*P* < 0.05). On day 10 (Table 2), when compared with the control, the PVA/NOCC-implanted rats showed significant alterations in the levels of platelets, eosinophils, GGT, ALP, urea, and potassium, while the HAM-implanted rats exhibited alterations in the levels of monocytes, neutrophils, GGT, ALP, urea, and creatinine. On day 15 (Table 3), when compared with the control, PVA/NOCC implantation induced significant alterations in the levels of potassium, urea, ALP, ALT, AST, GGT, hemoglobin, erythrocytes, WBC, platelets, creatinine, and neutrophils (*P* < 0.05); on the other hand, HAM implantation led to changes in the levels of potassium, creatinine, total protein, eosinophil, ALP, ALT, AST, GGT, hemoglobin, erythrocytes, platelets, and neutrophils (*P* < 0.05).

**Figure 2:**
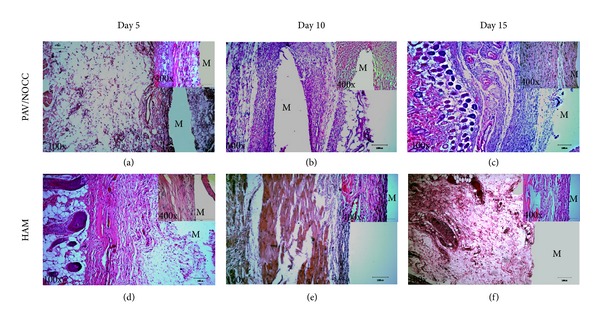
Histological sections and H&E staining of the PVA hydrogel and HAM after 5, 10, and 15 days of subcutaneous implantation. “M” indicates the implantation sites, 100x (low) and 400x (high). The high magnification photograph indicates the inflammatory infiltrates after implantation at variable time points.

Figures 1(a)–1(d) show HAM and PVA/NOC scaffolds, site of implantation, method of scaffold implantation, and site appearance after implantation, respectively. Consistent with the aforementioned findings, the histological staining images (Figure 2) showed inflammatory cells infiltration and distribution of leukocytes in the exudates in the tissue area where the test materials were implanted.

**Figure 3:**
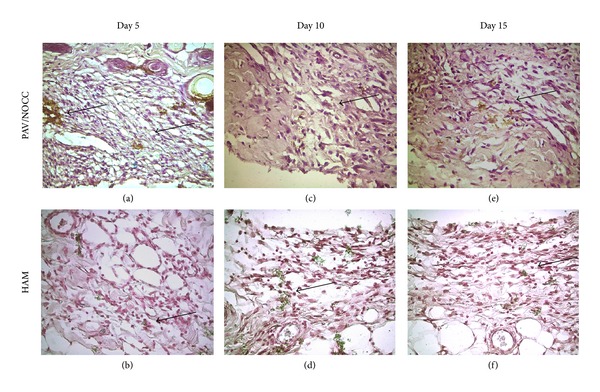
Immunohistology staining for CD 68 of the PVA hydrogel and HAM after 5, 10, and 15 days of subcutaneous implantation, 400x. Briefly, the samples were rinsed with phosphate buffered saline (PBS) and fixed with methanol for 15 min. Then, the samples were treated with 0.03% hydrogen peroxide for 5 min and incubated with mouse anti-rabbit anti-CD 68 antibody for 30 min at 1 : 100 dilutions and then with peroxidase-labeled polymer conjugated to goat anti-mouse immunoglobulin for another 30 min. The expression of CD 68 has been indicated by arrow (100x).

Immunohistochemical analysis was performed to assess whether the blood profile and histological findings were correlated with tissue localization of CD 68 expression, which indicates the presence of macrophages. High-intensity CD 68 staining showed that the immunological reactions around PVA/NOCC implants were high, when compared with those around HAM implants (Figure 3). Although the nature of the initial tissue response to both PVA/NOCC and HAM implants during the first 5 days (Figures 3(a) and 3(b)) of implantation was relatively similar, the amount of exudates and the number of acute cells were different on other time points such as days 10 and 15 (Figures 3(c), 3(d), 3(e), and 3(f)).

## 4. Discussion 

The degree of biocompatibility of a material depends on its properties such as shape, size, surface chemistry, porosity, sterility, contact duration, and degradation [2, 12]. Implantation of a biomaterial is mostly associated with an acute inflammatory response or sublethal toxicity. The inflammatory reactions might induce infiltration of polymorphonuclear leukocytes, macrophages, fibroblasts, and lymphocytes, and both acute inflammatory response and sublethal toxicity may last for days to weeks, depending on the type of implant material [13]. When compared with PVA/NOCCC, HAM, which is found in the innermost lining of the placenta, has low immunogenicity [14] as well as antiadhesive, anti-inflammatory, and antimicrobial properties [1, 15, 16]. Furthermore, HAM is a well-known biomaterial used in many clinical and research applications and is a suitable candidate for comparison because, in addition to being safe, it generally elicits little or no inflammatory response [17].

To our knowledge, this is the first report to examine the acute toxicity of PVA/NOCC in rats at three different time points of 5, 10, and 15 days with respect to hematological, biochemical, histological, and immunohistochemistry parameters. The site of scaffold implantation is subcutaneous, which involves different type of cells like fibroblast and macrophage. Therefore, biodegradation of natural or synthetic biomaterials is presumed to expel some primary or secondary compounds which may induce acute or chronic inflammatory reactions in the host tissue which may indirectly affect major organs like liver and kidney and so forth through the systemic blood circulation. Therefore, thorough complete blood count, liver function test, and kidney function test are important to determine in vivo biocompatibility of this biomaterial in short term implantation period is completely justifiable.

Histology results indicated that implanted materials may have provoked recruitment of inflammatory mediators to the site of implantation. Interestingly, host cell infiltration was notably high in the PVA/NOCC-implanted rats, when compared with that of HAM-implanted rats. This finding is in accordance with the results reported in previous studies, which demonstrated that PVA implantation induced some acute tissue responses in vivo [18, 19].

It has been suggested in previous reports that the use of steroids may reduce the inappropriate responses induced by implantation materials. In a study in which dexamethasone was incorporated into a PLGA/PVA composite, the release of dexamethasone from the composite was found to adequately control the acute inflammatory response for 1 month [19]. Furthermore, it was reported that, with some modifications, this control could be sustained for a period of up to 3 months [11]. It has been proposed that the initial rapid release of dexamethasone simply helps in delaying, rather than suppressing, the inflammation that occurs due to foreign body reaction [18, 19]. Another study indicated that PVA/alginate sample (5% alginate) could improve tissue compatibility by eliciting mild foreign body reactions during acute-phase subcutaneous implantation [13]. In the present study, contrary to our expectation, fabrication of NOCC with PVA did not ameliorate the acute tissue response induced by PVA. In addition to the complete blood count and histology analysis, the activation of the inflammatory cells was confirmed using immunohistochemistry. The extent of acute cell reactions induced by HAM implantation was low, when compared with that induced by PVA hydrogel, suggesting that changes in acute foreign body reactions could be related to the biodegradation properties of the implanted material. In general, acute inflammatory response to biomaterials is triggered once these materials are opsonized by host proteins such as IgG and complement cascade. These receptors may also play a role in the activation of the attached neutrophils or macrophages [13]. The limitation of the present study is that the degree of inflammation was not examined in long term. Although some degree of tissue response was noted in the PVA/NOCC-implanted group, whether such responses were significantly higher than those observed in the control group was not established. A recent study by our group revealed that tissue responses were negligible in rats implanted with nanohydroxyapatite- (HA-) PVA/NOCC bilayered scaffold, indicating that the compatibility of PVA/NOCC was improved when used as a bilayered scaffold [20]; however, the reason for these changes was elusive.

## 5. Conclusion

The results of the present study demonstrated PVA/NOCC signs of toxicity. With regard to biocompatibility, although PVA/NOCC implantation produced low toxic tissue response, it is yet to be determined whether such inflammatory reaction is clinically significant. Therefore, further studies are necessary to investigate such biomaterials because of some possible concerns in their use.

## Figures and Tables

**Table 1:** Differences in the level of some biochemical indicators among groups of animals implanted with PVA/NOCC and HAM and control on the 5th day of implantation (mean ± SD).

Parameter	Unit	PVA/NOCC	HAM	Control (no implants)
Potassium	mmol/L	4.76 ± 0.58∗	4.20 ± 0.45	4.13 ± 0.29
Urea	mmol/L	6.63 ± 0.61∗	6.41 ± 0.87∗	5.15 ± 0.57
Creatinine	mmol/L	35.00 ± 2.68∗	24.14 ± 3.57	27.33 ± 6.22
Total protein	g/L	62.00 ± 2.42∗	63.57 ± 1.65	64.50 ± 3.02
Albumin	g/L	9.50 ± 1.37	10.71 ± 0.91	10.17 ± 1.17
Globulin	g/L	52.38 ± 2.53	52.71 ± 1.44	54.33 ± 2.50
ALP	IU/L	214.75 ± 31.05∗	239.71 ± 19.65∗	310.83 ± 57.47
ALT	IU/L	49.00 ± 3.27∗	55.43 ± 5.40∗	71.00 ± 4.24
AST	IU/L	156.25 ± 22.99∗	167.00 ± 15.74∗	189.67 ± 13.71
GGT	IU/L	4.00 ± 0.68∗	3.29 ± 0.469∗	2.00 ± 0.00
Hemoglobin	g/L	134.38 ± 8.96∗	131.43 ± 8.72	122.83 ± 2.93
Erythrocytes	10^∧^12/L	6.68 ± 1.28	6.96 ± 0.55	6.23 ± 0.51
WBC	10^∧^9/L	13.45 ± 1.86∗	10.04 ± 1.21	11.58 ± 1.62
Platelet	10^∧^9/L	634.00 ± 247.24	536.43 ± 80.97	570.83 ± 161.97
Neutrophil	10^∧^9/L	2.31 ± 0.55∗	2.41 ± 0.39∗	0.86 ± 0.27
Lymphocyte	10^∧^9/L	9.07 ± 3.03	6.89 ± 0.841∗	10.56 ± 5.84
Monocyte	10^∧^9/L	1.14 ± 0.42∗	0.56 ± 0.066	0.64 ± 0.40
Eosinophil	10^∧^9/L	0.005 ± 0.018∗	0.07 ± 0.01∗	0.15 ± 0.05

*P < 0.05.

**Table 2:** Differences in the level of some biochemical indicators among groups of animals implanted with PVA/NOCC and HAM and control on the 10th day of implantation (mean ± SD).

Parameter	Unit	PVA/NOCC	HAM	Control (no implants)
Potassium	mmol/L	5.39 ± 1.87∗	5.19 ± 1.35	4.13 ± 0.29
Urea	mmol/L	6.28 ± 0.85∗	6.29 ± 0.58∗	5.15 ± 0.57
Creatinine	mmol/L	29.25 ± 5.20	20.50 ± 2.55∗	27.33 ± 6.22
Total protein	g/L	62.38 ± 4.12	64.71 ± 3.66	64.50 ± 3.02
Albumin	g/L	9.88 ± 1.19	11.25 ± 1.59	10.17 ± 1.17
Globulin	g/L	52.50 ± 4.21	55.00 ± 4.30	54.33 ± 2.50
ALP	IU/L	225.38 ± 49.17∗	227.75 ± 38.09∗	310.83 ± 57.47
ALT	IU/L	67.70 ± 20.87	68.13 ± 14.82	71.00 ± 4.24
AST	IU/L	216.57 ± 46.29	162.75 ± 33.98	189.67 ± 13.71
GGT	IU/L	2.80 ± 0.51∗	2.88 ± 0.61∗	2.00 ± 0.00
Hemoglobin	g/L	123.75 ± 18.45	127.63 ± 21.07	122.83 ± 2.93
Erythrocytes	10^∧^12/L	6.63 ± 0.78	6.82 ± 1.12	6.23 ± 0.51
WBC	10^∧^9/L	11.36 ± 4.76	10.55 ± 6.14	11.58 ± 1.62
Platelet	10^∧^9/L	474.00 ± 103.15∗	554.00 ± 103.26	570.83 ± 161.97
Neutrophil	10^∧^9/L	1.26 ± 0.56	1.84 ± 0.55∗	0.86 ± 0.27
Lymphocyte	10^∧^9/L	8.71 ± 5.82	7.21 ± 2.45	10.56 ± 5.84
Monocyte	10^∧^9/L	0.59 ± 0.36	0.29 ± 0.19∗	0.64 ± 0.40
Eosinophil	10^∧^9/L	0.07 ± 0.06∗	0.15 ± 0.07	0.15 ± 0.05

*P < 0.05.

**Table 3:** Differences in the level of some biochemical indicators among groups of animals implanted with PVA/NOCC and HAM and control on the 15th day of implantation (mean ± SD).

Parameter	Unit	PVA/NOCC	HAM	Control (no implants)
Potassium	mmol/L	4.51 ± 0.47∗	4.76 ± 0.78∗	4.13 ± 0.29
Urea	mmol/L	7.37 ± 1.06∗	5.12 ± 0.76	5.15 ± 0.57
Creatinine	mmol/L	34.23 ± 5.78∗	33.20 ± 3.46∗	27.33 ± 6.22
Total protein	g/L	62.57 ± 1.874	59.80 ± 1.64∗	64.50 ± 3.02
Albumin	g/L	10.86 ± 1.27	10.60 ± 0.82	10.17 ± 1.17
Globulin	g/L	51.71 ± 2.42	49.20 ± 2.38	54.33 ± 2.50
ALP	IU/L	243.86 ± 63.19∗	128.00 ± 12.88∗	310.83 ± 57.47
ALT	IU/L	58.71 ± 7.48∗	50.40 ± 1.67∗	71.00 ± 4.24
AST	IU/L	175.57 ± 12.01∗	147.20 ± 13.57∗	189.67 ± 13.71
GGT	IU/L	3.00 ± 0.00∗	3.10 ± 0.02∗	2.00 ± 0.00
Hemoglobin	g/L	136.86 ± 6.09∗	133.60 ± 14.85∗	122.83 ± 2.93
Erythrocytes	10^∧^12/L	7.43 ± 0.57∗	7.07 ± 0.80∗	6.23 ± 0.51
WBC	10^∧^9/L	14.93 ± 3.09∗	12.14 ± 0.72	11.58 ± 1.62
Platelet	10^∧^9/L	758.86 ± 69.69∗	702.80 ± 89.25∗	570.83 ± 161.97
Neutrophil	10^∧^9/L	2.23 ± 0.91∗	1.52 ± 0.26∗	0.86 ± 0.27
Lymphocyte	10^∧^9/L	9.99 ± 2.92	8.60 ± 0.91	10.56 ± 5.84
Monocyte	10^∧^9/L	0.73 ± 0.13	0.68 ± 0.05	0.64 ± 0.40
Eosinophil	10^∧^9/L	0.15 ± 0.02	0.10 ± 0.02∗	0.15 ± 0.05

*P < 0.05.
